# Human Leukocyte Antigen Profiles of Latin American Populations: Differential Admixture and Its Potential Impact on Hematopoietic Stem Cell Transplantation

**DOI:** 10.1155/2012/136087

**Published:** 2012-11-18

**Authors:** Esteban Arrieta-Bolaños, J. Alejandro Madrigal, Bronwen E. Shaw

**Affiliations:** ^1^Clinical Research Group, The Anthony Nolan Research Institute, Royal Free & University College Medical School, London NW3 2QG, UK; ^2^University College London Cancer Institute, London WC1E 6DD, UK; ^3^Centro de Investigaciones en Hematología y Trastornos Afines (CIHATA), Universidad de Costa Rica, 11501-2060 San José, Costa Rica; ^4^Haemato-Oncology Research Unit, Division of Molecular Pathology, The Institute of Cancer Research, London SM2 5NG, UK

## Abstract

The outcome of hematopoietic stem cell transplantation (HSCT) is shaped by both clinical and genetic factors that determine its success. Genetic factors including human leukocyte antigen (HLA) and non-HLA genetic variants are believed to influence the risk of potentially fatal complications after the transplant. Moreover, ethnicity has been proposed as a factor modifying the risk of graft-versus-host disease. The populations of Latin America are a complex array of different admixture processes with varying degrees of ancestral population proportions that came in different migration waves. This complexity makes the study of genetic risks in this region complicated unless the extent of this variation is thoroughly characterized. In this study we compared the HLA-A and HLA-B allele group profiles for 31 Latin American populations and 61 ancestral populations from Iberia, Italy, Sub-Saharan Africa, and America. Results from population genetics comparisons show a wide variation in the HLA profiles from the Latin American populations that correlate with different admixture proportions. Populations in Latin America seem to be organized in at least three groups with (1) strong Amerindian admixture, (2) strong Caucasian component, and (3) a Caucasian-African gradient. These results imply that genetic risk assessment for HSCT in Latin America has to be adapted for different population subgroups rather than as a pan-Hispanic/Latino analysis.

## 1. Introduction

Hematopoietic stem cell transplantation (HSCT) is a curative therapy used for the treatment of malignant and nonmalignant hematologic diseases, congenital immune deficiencies, solid tumors, and metabolic diseases [1]. Its outcome is shaped not only by clinical factors [2], but also by the genetics of the patient-donor pair [3]. Apart from the normal compatibility defined by the human leukocyte antigen (HLA) system [4, 5], variation in several genetic systems is thought to have an impact on the complications experienced by patients that undergo this procedure [6]. 

Graft-versus-host disease (GVHD) is a major complication affecting the success of the transplant and the survival of the patients. Despite the fact that most transplants are performed with high levels of compatibility in terms of HLA, a significant proportion of these transplants is affected by GVHD. Apart from clinical factors [7], a genetic component for GVHD other than HLA has been pointed out as responsible for the occurrence of GVHD in 10/10 HLA compatible patient-donor pairs [8, 9]. Moreover, an ethnicity-driven risk of suffering GVHD after HSCT has been identified [10, 11]. However, these studies focused on “island” populations and broader populations with low admixture proportions, and further studies in admixed populations are lacking.

Latin America is a region where the most dramatic human migrations have taken place, from the early northeastern Asian bands of hunter-gatherers that conquered the last continent humanity had expanded to [12], through the 16th and 17th centuries European colonization and bringing of sub-Saharan African (SSA) slaves [13], to the latest waves of immigrants from all over the world in the last two centuries [14]. This complex population history makes present Latin American Populations (LAP) possibly the most ethnically diverse on the planet. This genetic diversity is thus likely to impact the effect of genetics on HSCT and hence it is necessary to understand it in order to be able to interpret genetic association studies in this and other medical fields.

In this study, we used population genetics tools to compare the HLA profiles of 31 LAP and 61 ancestral populations in order to characterise their diversity and classify them according to their genetic makeup.

## 2. Materials and Methods

### 2.1. Population Samples

A selection of 92 populations from Latin America, Iberia, Italy, and sub-Saharan Africa with available DNA-based typing data for HLA-A and HLA-B allele groups was made and their details are shown in Table 1. Of these, 31 LAP were defined as populations living in this region that were not classed as Amerindian. Population samples from LAP that have emigrated to the USA and Spain were also included in the analyses.

**Figure 1:**
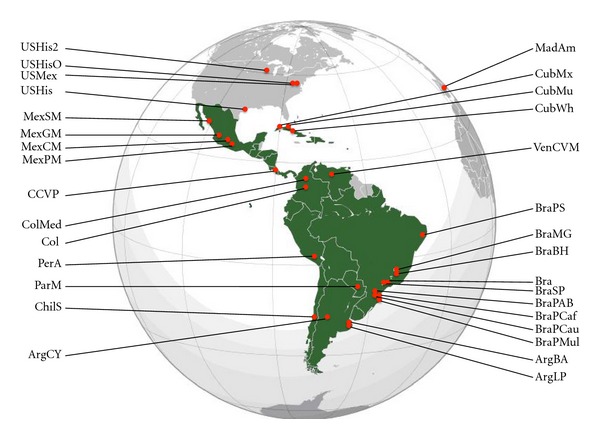
Map showing the approximate location of the LAP included in the study.

The remaining 61 populations are native population samples from the three ancestral regions that have contributed majorly to the Latin American gene pool: Amerindians (22 populations), Caucasians from Europe (Iberians and Italians, 19 populations), and SSA (20 populations). In the Caucasian population group, a sample of Italians was selected to complement the Iberian populations in view of the important immigration from this country into some LAP. In total, the population array included 384,446 chromosomes. HLA frequency data was extracted from journal articles and/or the Allele Frequencies database website [15]. The approximate geographic location for the LAP is shown in Figure 1.

### 2.2. Database Construction

A database containing the frequencies for 47 HLA-A and HLA-B allele groups from the 92 populations that were selected was built. When the available data were at high resolution, the data were reduced to two-digit allele groups. The database was constructed on the Multi-Variate Statistical Package (MVSP, Kovach Computing Services, Anglesey, Wales) and was independently checked for accuracy.

### 2.3. Population Comparisons

The HLA-A and HLA-B profiles of the 92 populations were analysed by clustering analysis and Principal Coordinates Analysis (PCO), both based on Euclidean distances. The clustering analysis was performed dually and dendrograms were generated for both analyses. The clustering method was based on minimum variance of squared Euclidean distances with a randomized input order. The Eigenanalysis for the PCO was performed at an accuracy of 1E-7 and axes were extracted according to Kaiser's rule [66].

Additionally, three ancestry-specific HLA allele groups were compared between population subgroups in order to illustrate the relative contribution of each ancestral population across LAP. 

## 3. Results

### 3.1. Clustering Analysis

**Figure 2:**
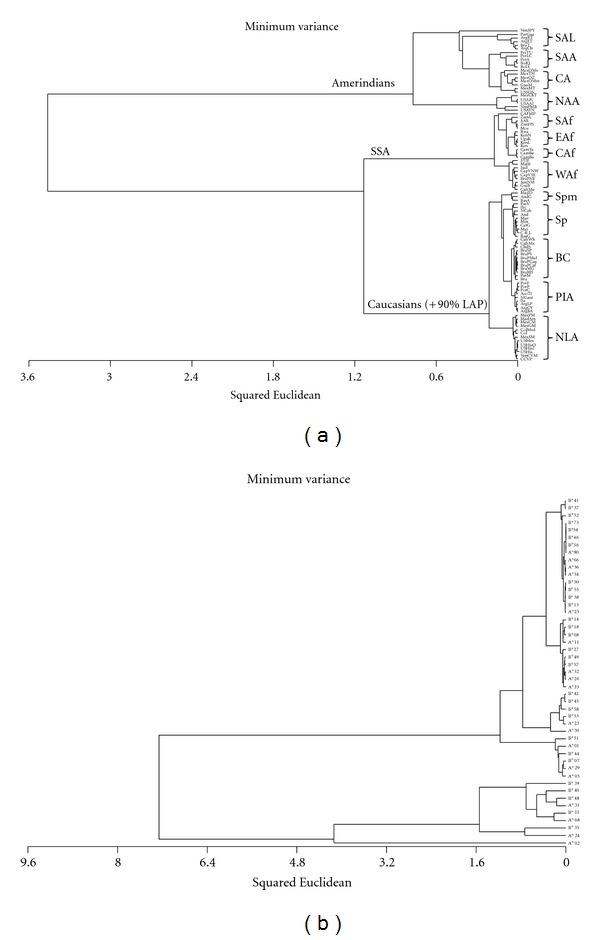
Cluster analysis based on 47 HLA-A and HLA-B allele group frequencies among 31 LAP and 61 ancestral populations. (a) Dendrogram showing the clustering of the 92 populations. (b) Dendrogram showing the dual-clustering of HLA allele groups in the dataset. SSA: Sub-Saharan Africans; SAL: South American Lowlanders; SAA: South American Andeans; CA: Central Americans; NAA: North Americans and Alaskans; SAf: Southern Africans; EAf, Eastern Africans; CAf: Central Africans; WAf: Western Africans; Spm: Spanish minorities; Sp: Spanish; BC: Brazilians and Cubans; PIA: Portuguese, Italians, and Argentinians; NLA: Northern Latin Americans.

A dendrogram based on squared Euclidean distances was generated by the comparison of 47 HLA-A and HLA-B allele group frequencies present in the 61 ancestral populations and the 31 LAP. The results for this analysis are shown in Figure 2(a). The first split is between the Amerindian cluster and the Caucasians and SSA, which is consistent with higher differentiation of these populations. The next split is between the SSA and the Caucasian and most of the LAP. 

A closer look at the clusters shows that there is a correlation between the geographic location of the ancestral populations and the branching within the clusters. Within the Amerindian cluster, 4 groups form a South American lowland group, a South American Andean group, a Central American group, and a more distinct North American-Alaskan group. A similar correlation is seen within the SSA cluster: western Africans split from the southern, eastern and central African populations. Some of the LAP cluster with the Amerindians, such as the Peruvian mestizos from Arequipa, or with the SSA, as the Cuban Mulattos and the Afro-Brazilians from Paraná. However, 90% of the LAP cluster within a distinct group which includes the Iberians and Italians.

The LAP-Iberian+Italian cluster splits further in distinct subgroups. Most of the Spanish populations, and minority populations from Spain, cluster in their own groups. Also, there is a broad group that clusters all of the remaining Brazilian and Cuban populations, and another one that clusters the Portuguese, Italian, and Argentinians from La Plata, the region of Cuyo, and Buenos Aires. Finally, the last cluster includes the admixed populations from Mexico, Colombia, Venezuela, Costa Rica, as well as the South American immigrants to Madrid and the Mexican and pan-Hispanic samples from the United States.

A dual-clustering method was applied to the dataset in order to identify the groups of alleles that are most variable between the populations. The results from this analysis are shown in Figure 2(b). Clusters of signature ancestry markers can be identified, such as frequent Amerindian input allele groups (HLA-A*68, -B*15, -A*31, -B*48, -B*40, and -B*39), frequent Iberian and Italian Caucasian markers (HLA-A*03, -A*29, -B*07, -B*44, -A*01, and-B*51), and frequent alleles that are evidence of SSA genetic input (HLA-A*30, -A*23, -B*53, -B*58, -B*45, and -B*42). 

### 3.2. Principal Coordinates Analysis

**Figure 3:**
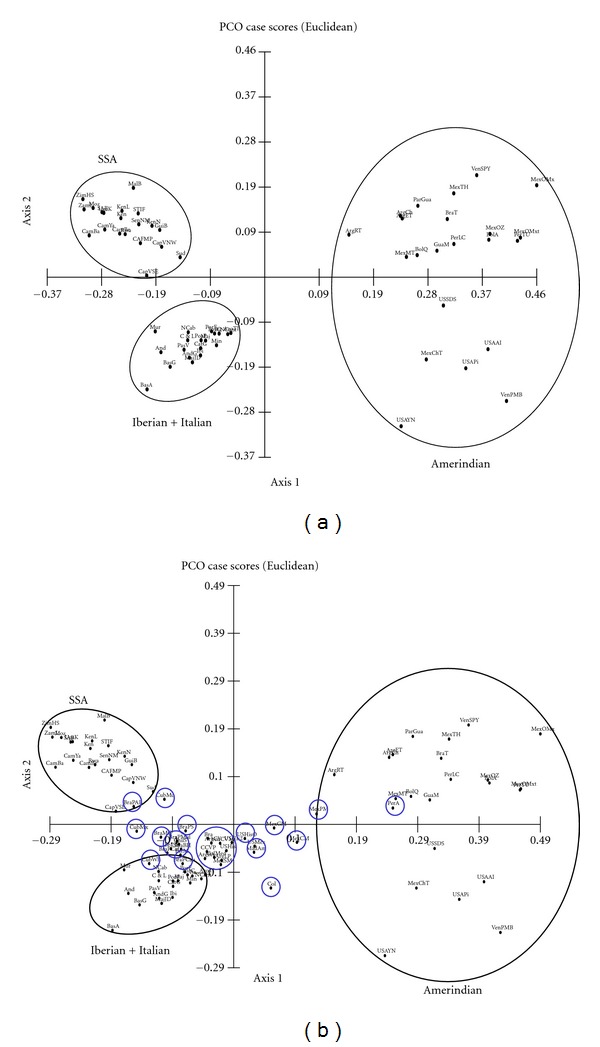
Principal coordinates analysis (PCO) based on the frequencies of 47 HLA-A and HLA-B allele groups in 31 LAP and 61 ancestral populations. (a) PCO map of the first 2 principal components (57.7% cumulative variance) for 61 ancestral populations from sub-Saharan Africa (SSA), America, and Europe. (b) PCO map showing the first 2 principal components (56.7% cumulative variance) for 31 LAP (blue) and 61 ancestral populations.

The results from the PCO are shown in Figure 3. Firstly, the ancestral populations (Figure 3(a)) show a clear location. The first PC correlates with the Amerindian-non-amerindian split seen in the cluster analysis, whereas the second split (SSA-Caucasians) correlates with the second PC. Amerindian populations show a much more dispersed array and higher distances from the Caucasians and the SSA, which is in accordance with their genetic history being shaped by the colonization of the last continent after the out-of-Africa migrations and successive bottlenecks in this process [67]. Interestingly, SSA that have been in closer genetic and geographic contact with the Caucasians, such as the Sudanese and the Cape Verdeans, are closer to these populations, whereas the southernmost Africans lie on the upper left extreme of the PCO map. Likewise, the North American Amerindians tend to be closer to the Alaskan Natives, who have been shown to be genetically different from the Amerindians of more southern regions [30]. 

When the LAP are included in the analysis (Figure 3(b)), the results show that LAP are located on a wide arch that connects the three ancestral populations. This arch stretches from the Peruvian mestizos from Arequipa, which appear deep into the Amerindian region, to the Afro-Brazilians from Paraná, which lie on the periphery of the SSA cluster. In between these populations there is a spectrum of locations for the remaining LAP. It is clear that there are two major regions: one that includes the LAP that lie between the Iberian and Italian populations and the Amerindian region and the others, which lie between the Caucasians and the SSA populations. Moreover, the first group seems to be divided in two subregions: one that clusters populations that lie closely to the Caucasian group (from the Argentinians from Buenos Aires to the Hispanic samples from the US), and the other (from the Mexican population from the US up to the Peruvians from Arequipa) which is dragged more intensely towards the Amerindians. The SSA component in these populations seems to be reduced, although not absent (see below). On the other hand, the populations that lie between the Caucasians and the SSA samples also cluster closely to the Iberians and Italians, but show a gradient towards the SSA cluster. This group is composed mainly by Brazilian and Cuban populations. 

A few populations seem to cluster closely enough to the ancestral populations to be considered part of those clusters. This is the case of the Cuban Mulattos and the Afro-Brazilians from Paraná, the Cuban Whites, the Brazilian Caucasians from Paraná, and the Peruvians of Arequipa. In turn, it must be noted that a population classified as Amerindian, the Argentinian Toba from the city of Rosario, show significant Caucasian admixture and, consequently, lie closer to the admixed Mexicans than the Peruvians do.

### 3.3. Specific Ancestry Markers

**Figure 4:**
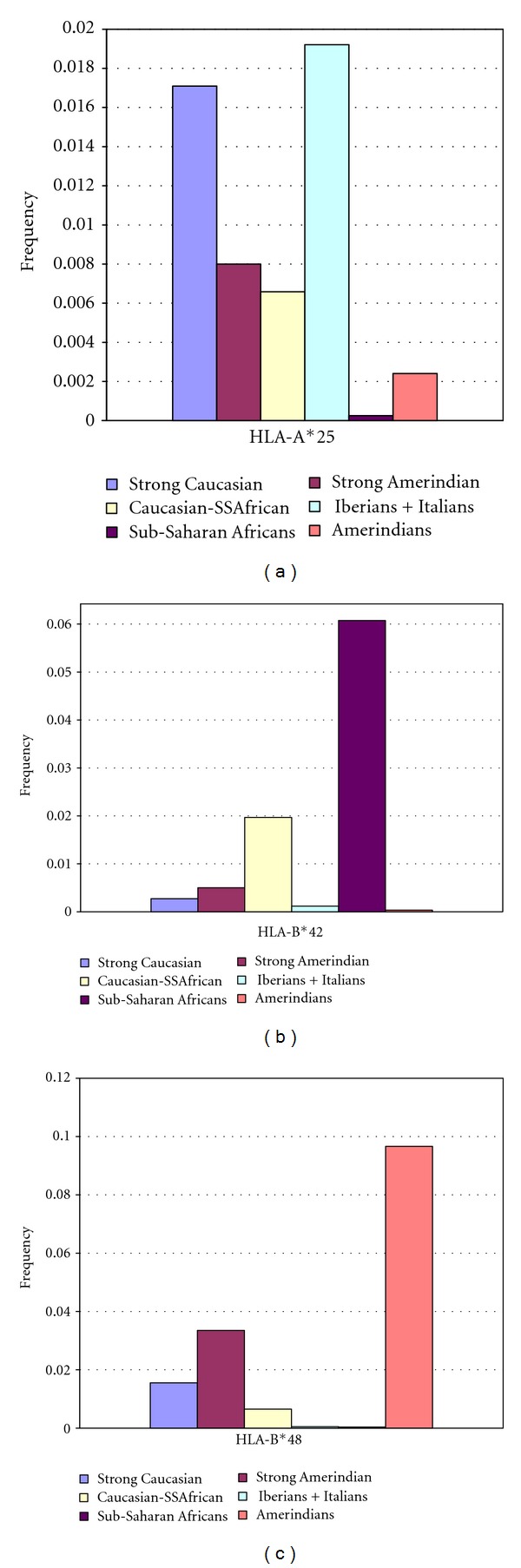
Frequency of ethnic-specific HLA allele groups among three subgroups of LAP and the ancestral populations. (a) Frequency of HLA-A*25 allele group as a Caucasian marker. (b) Frequency of HLA-B*42 allele group as a Sub-Saharan African (SSA) marker. (c) Frequency of HLA-B*48 as an Amerindian marker.

To further illustrate the differential admixture patters present in LAP based on their HLA profile, 3 allele groups which are present in one of the ancestral populations and absent or nearly absent in the other two (HLA-A*25, -B*42, and -B*48) were selected in order to evaluate their frequency among the LAP groups (Figure 4). As seen in Figure 4(a), HLA-A*25, a common allele group in western Europe and virtually absent in Amerindian and SSA populations, is present more frequently in the admixed populations with strong Caucasian component (i.e., those that lie closest to the Caucasians on the Caucasian-Amerindian axis of the PCO). Interestingly, some of the ancestral populations classified as Amerindian show evidence of Caucasian admixture as demonstrated by the presence of HLA-A*25 alleles in their gene pool. 

Figure 4(b) shows the frequency of the SSA allele group HLA-B*42 in the 3 LAP subgroups. It is evident that these alleles are much more frequent in these populations (mainly, Brazilian and Cuban) than in the rest. However, HLA-B*42 alleles are not absent from other LAP, which may have lower levels of SSA admixture.

Finally, Figure 4(c) shows the average frequency of HLA-B*48 alleles. This group, common in Amerindians and nearly absent from SSA and Iberian and Italian populations, is more strongly represented in the populations that form the bridge between the Amerindian and Caucasian regions in the PCO.

## 4. Discussion

The results obtained after comparing 47 HLA-A and HLA-B allele groups among the LAP and their ancestral populations show that there is widespread variation between the genetic profiles of these admixed or exported populations. In the cluster analysis it is clear that most LAP have substantial Caucasian components, with the exception of some populations such as the Peruvians from Arequipa or the Afro-Brazilians from Paraná. This is in agreement with the uneven process of population replacement and the collapse of many Native American groups that took place throughout the continent. 

However, PCO analysis showed that most LAP sit on a wide admixture arch that approaches the ancestral clusters. A few populations fall very close to or in the ancestral clusters, but most are scattered in intermediate regions. Interestingly, population samples that are likely to be a mixture of several LAP, such as those of the USA Hispanic immigrants and the Ibero-American expatriates in Madrid, sit in the center of the distribution. In fact, the heterogeneity of the Hispanic population in the US has been described using other markers [68, 69], showing differential admixture patterns between areas that have received mostly Mexican immigration and those that are predominantly colonized by Caribbean islanders from Cuba and Puerto Rico. In agreement with this, US Mexicans lie slightly closer to the Amerindian side on the PCO and locate between the Mexican populations from the center and the sample from the northern state of Sinaloa. This further illustrates the heterogeneity of the Mexican populations, where a stronger Caucasian component is preserved in the north of the country [43], while the US Mexicans are likely to be a combination of northern and southern Mexican populations.

The stronger Caucasian component in some LAP can be attributed to recent European migration [14], such as that of urban populations from Argentina and some Brazilian populations, or to relatively stronger Caucasian proportions generated at colonial times in areas where Amerindian populations were low at the time of the arrival of Europeans, which is thought to be the case of the Costa Rican Central Valley and the Colombians from Medellin [70, 71].

On the Caucasian-SSA axis of admixture, several Brazilian and Cuban populations can be found. It seems that for these populations, Amerindian admixture is very low or absent. This has been noted by others [72] and it is argued that a dual admixture model is more likely to describe the patterns seen in these populations as opposed to a triple admixture model identified for other LAP. Although not included in our analysis because of the lack of molecular HLA data, serological HLA data from Panama [73] and Puerto Rico [74] suggest that these populations are likely to join this group, whereas the data from Uruguay suggest that its major population would cluster with the strong Caucasian component group [75].

Our study is limited by both the availability of population data and the need to use HLA allele group data for comparison as opposed to high-resolution allele frequencies or haplotype frequencies. It is likely that an analysis of high-resolution frequencies would give finer results, but it would seriously diminish the number of populations that can be included in the analysis. However, the use of 47 allele groups from the most polymorphic genes in the human genome gives robustness to the analysis.

The effect of ethnicity on complications after HSCT has been suspected for many years [76, 77] but some studies have not shown such association [78]. Hence, there is growing interest in unraveling the genetic-ethnic component of GVHD in HLA-compatible HSCT. Currently, there is a project within the International Histocompatibility Working Group that aims at analyzing the risk of GVHD after HSCT in unrelated donor pairs according to the ethnic origin of both patients and donors, based on previous findings in sibling transplantations in isolated and general populations of certain countries [10]. Preliminary results in a cohort of unrelated transplants showed that Hispanic pairs have high risks of mortality and acute GVHD (grades III-IV) only second to African American pairs. Moreover, Hispanic-Hispanic pairs had the highest risk of relapse [79]. Both analyses were carried out having Asian/Pacific (mostly Japanese) ethnically matched pairs as the reference group. These findings suggest that ethnic heterogeneity in the Hispanic population may be playing a role on the risk of complications after HSCT, and the complexity of the admixture patterns illustrated in this study and others is likely to account for much of this variation. Also, ethnicity has been associated with other complications after HSCT such as chronic GVHD [80]. Moreover, an increased risk of complications has been reported specifically for Hispanic groups in North America when compared to other ethnicities in terms of survival [81] and treatment failure [82]. 

It is likely that the evidence for differential outcome in different ethnic groups could be explained, at least in part, by differences in allele frequencies in genes that are relevant to the immune response and that show variable interethnic polymorphism, such as the cytokine genes [83]. Moreover, polymorphisms in other genes such as those that intervene in drug metabolism or drug targets may play a role in the way patients from different ethnicities respond to treatment in HSCT, especially in admixed populations [84, 85]. 

LAP show widespread variation in their genetic profiles, and this complicates genetic association studies made in these populations. There is noticeable variation not only between regions and countries, but also between areas of the same country [43, 86]. Furthermore, the presence of minority populations of different ethnic composition adds to the complexity of population stratification in Latin America. Additionally, many populations remain to be studied. If an ethnic component is to be used as one prognostic factor affecting the risk of complications after HSCT, the application of this concept in Latin American populations will have to take into account the great diversity found among the different populations derived from this region and the different population subgroups generated by different admixture histories. Consequently, there is need of a more detailed understanding of the genetic profiles of the LAP, in order to be able to accurately stratify genetic risk in HSCT.

It is also important that a better definition of individual ancestry in LAP is reached in view of the evident limitations of both self-reported [87] and researcher-assigned ethnicities [41, 88]. To this purpose, the use of a more objective assignment based on ancestry markers [69] is likely to increase the accuracy of the information derived from these studies. Hopefully, a finer characterization of the risk of complications after HSCT in LAP will help foresee these complications and increase the access and success of transplantation in these populations.

## Figures and Tables

**Table 1:** Summary and details of the populations included in the analyses.

Code	Population	Size (2*n*)	Reference
Amerindians			
ArgCh	Argentinian Chiriguano	108	[15]
ArgET	Argentinian Eastern Toba	270	[16, 17]
ArgRT	Argentinian Toba from Rosario	172	[15]
BolA	Bolivian Aymara	204	[18]
BolQ	Bolivian Quechua	160	[19]
BraT	Brazilian Terena	120	[20]
GuaM	Guatemalan Maya	264	[21]
MexChT	Mexican Tarahumara from Chihuahua	88	[22]
MexMT	Mexican Tarasco from Michoacán	260	[23]
MexOMx	Mexican Mixe from Oaxaca	110	[24]
MexOMxt	Mexican Mixtec from Oaxaca	206	[24]
MexOZ	Mexican Zapotec from Oaxaca	180	[24]
MexTH	Mexican Teenek from Huasteca region	110	[25]
ParGua	Paraguayan Guaraní	80	[26]
PerLC	Peruvian Lama	166	[15]
PerTU	Peruvian Uro	210	[27]
VenPMB	Venezuelan Bari	110	[28]
VenSPY	Venezuelan Yucpa	146	[29]
USAYN	Alaska Yupik Natives	504	[30]
USAAI	Arizona Gila River Indian	984	[31]
USAPi	Arizona Pima	200	[28]
USSDS	South Dakota Lakota Sioux	604	[32]

LAP			
ArgBA	Argentinians from Buenos Aires	2,432	[15]
ArgCY	Argentinians from Cuyo Region	840	[15]
ArgLP	Argentinians from La Plata	200	[15]
Bra	Brazilians	216	[28]
BraBH	Brazilians from Belo Horizonte	190	[33, 34]
BraMG	Brazilians from Minas Gerais	2,000	[15]
BraPAB	Afro-Brazilians from Paraná	154	[35]
BraPCaf	Brazilian Cafuzo from Paraná	638	[35]
BraPCau	Brazilian Caucasian from Paraná	5,550	[35]
BraPMul	Brazilian Mulatto from Paraná	372	[35]
BraPS	Brazilians from Pernambuco State	202	[36]
BraSP	Brazilians from Sao Paulo	478	[15]
CCVP	Costa Ricans from the Central Valley	364	[37]
ChilS	Chileans from Santiago	140	[15]
Col	Colombians	1,122	[38]
ColMed	Colombians from Medellin	1,852	[39]
CubMx	Cubans (mixed)	378	[40]
CubMu	Cuban Mulattos	84	[33, 34]
CubWh	Cuban Whites	140	[33, 34]
MadAm	Latin American immigrants in Madrid	346	[41]
MexGM	Mexicans from Guadalajara	206	[42]
MexCM	Mexicans from Mexico City	242	[43]
MexSM	Mexicans from Sinaloa	112	[43]
MexPM	Mexicans from Puebla	198	[43]
ParM	Paraguayans	100	[44]
PerA	Peruvians from Arequipa	336	[45]
USHis	US Hispanics	468	[15]
USHis2	US Hispanics	3,998	[46]
USHisO	US Hispanics	3,160	[47]
USMex	US Mexicans	1,106	[48]
VenCVM	Venezuelans from Caracas, Valencia, and Maracaibo	192	[15]

Iberians and Italians			
BasA	Basques from Arratia Valley	166	[49]
BasG	Basques from Guipuskoa	200	[50]
C&L	Castilians	3,880	[51]
CatG	Catalonians from Girona	176	[50]
And	Spanish from Andalucía	198	[15]
AndG	Spanish Gypsy from Andalucía	198	[15]
Ibi	Spanish from Ibiza	176	[52]
Maj	Majorcans	814	[52]
MajJD	Majorcans of Jewish descent	206	[52]
Min	Minorcans	188	[52]
Mur	Murcians	346	[53]
NCab	North Cabuernigo	190	[49]
NCant	North Cantabrians	166	[49]
PasV	Spanish from Pas Valley	176	[49]
AzoTI	Azoreans from Terceira Island	260	[15]
Ita	Italians	318,622	[54]
PorC	Portuguese from central Portugal	1,124	[15]
PorP	Portuguese from Porto	15,874	[15]
PorF	Portuguese from Faro	2,484	[15]

SSA			
CamBa	Cameroon Bamileke	154	[55]
CamBe	Cameroon Beti	348	[55]
CamYa	Cameroon Yaounde	184	[56]
CapVNW	Cape Verdeans from NW island	124	[57]
CapVSE	Cape Verdeans from SE island	124	[57]
CAFMP	Pygmy from the Central African Republic	72	[58]
GuiB	Guineans	130	[57]
Ken	Kenyans	288	[28]
KenL	Kenyans-Luo	530	[59]
KenN	Kenyans-Nandi	480	[59]
MalB	Mali Bandiagara	276	[59]
Moz	Mozambicans	500	[60]
Rwa	Rwandans	560	[61]
STIF	Sao Tome Islanders (Forro)	132	[62]
SenNM	Senegalese (Madenka)	330	[63]
SAB	Black South Africans	400	[64]
Sud	Sudanese	400	[15]
UgaK	Ugandan from Kampala	350	[65]
ZamL	Zambians from Lusaka	88	[59]
ZimHS	Zimbabwe Harare Shona	460	[28]

Total	92	384,446	
